# PGD: a machine learning-based photosynthetic-related gene detection approach

**DOI:** 10.1186/s12859-022-04722-x

**Published:** 2022-05-17

**Authors:** Yunchuan Wang, Xiuru Dai, Daohong Fu, Pinghua Li, Baijuan Du

**Affiliations:** State Key Laboratory of Crop Biology, College of Agronomic Sciences, Shandong Agricultural University, Tai’an, 271018 Shandong China

**Keywords:** Photosynthesis, Machine learning, Functional category, RNA-Seq, Ensemble learning

## Abstract

**Background:**

The primary determinant of crop yield is photosynthetic capacity, which is under the control of photosynthesis-related genes. Therefore, the mining of genes involved in photosynthesis is important for the study of photosynthesis. MapMan Mercator 4 is a powerful annotation tool for assigning genes into proper functional categories; however, in maize, the functions of approximately 22.15% (9520) of genes remain unclear and are labeled “not assigned”, which may include photosynthesis-related genes that have not yet been identified. The fast-increasing usage of the machine learning approach in solving biological problems provides us with a new chance to identify novel photosynthetic genes from functional “not assigned” genes in maize.

**Results:**

In this study, we proved the ensemble learning model using a voting eliminates the preferences of single machine learning models. Based on this evaluation, we implemented an ensemble based ML(Machine Learning) methods using a majority voting scheme and observed that including RNA-seq data from multiple photosynthetic mutants rather than only a single mutant could increase prediction accuracy. And we call this approach “A Machine Learning-based Photosynthetic-related Gene Detection approach (PGD)”. Finally, we predicted 716 photosynthesis-related genes from the “not assigned” category of maize MapMan annotation. The protein localization prediction (TargetP) and expression trends of these genes from maize leaf sections indicated that the prediction was reliable and robust. And we put this approach online base on google colab.

**Conclusions:**

This study reveals a new approach for mining novel genes related to a specific functional category and provides candidate genes for researchers to experimentally define their biological functions.

**Supplementary Information:**

The online version contains supplementary material available at 10.1186/s12859-022-04722-x.

## Introduction

Annotations of MapMan functional categories play a pivotal role in helping researchers identify candidate genes [1]. However, these functional categories are based on BLAST sequence similarity and protein domains from InterPro [2] and the Conserved Domain Database (CDD) [3], which assign genes that do not show high sequence similarity to Arabidopsis or contain no typical protein domains to the category of “not assigned”. In maize, approximately 9520 genes were sorted to the category of “not assigned” in the last version of MapMan Mercator 4, and we were curious whether there are other ways to help predict the potential functions of these genes.

Supervised machine learning approaches have recently been rapidly developed in the field of biological applications; e.g., AlphaFold, a novel machine learning approach, can predict protein structures with high accuracy [4]. This approach was recently successfully tested in maize to predict the functional annotations of non-homology-based genes [5]. Therefore, we believe it is worth testing the performance of a supervised machine learning approach in predicting the putative biological functions of these “not assigned” genes.

Photosynthesis plays a vital role in living organisms on our planet, powering our ecosystem by providing carbohydrates and oxygen [6]. The discovery of more photosynthesis-related genes could help broaden our knowledge of photosynthesis and further help to improve photosynthetic efficiency in plants, especially in crops. In recent decades, the discovery of photosynthesis-related genes in maize has mainly been based on sequence similarity with well-studied photosynthesis-related genes in Arabidopsis or rice [7], and the Photosynthetic Mutant Library (http://pml.uoregon.edu/pml_table.php), which uses forward genetics with Mu transposons, generated ~ 2100 mutants, corresponding to 95 identified genes involved in photosynthesis. All these studies greatly improved our understanding of photosynthesis; however, due to the complexity of photosynthesis, more new genes related to this important process need to be identified.

In this study, we implemented an ensemble based ML methods using a majority voting scheme, a supervised machine learning approach that integrates expression data and differential contrasts from six photosynthetic mutants to mine novel photosynthesis-related genes from the “not assigned” category of MapMan Mercator 4 in maize. The PGD approach generated a machine learning model with a high AUC-ROC (0.9649) and predicted 716 potential photosynthesis-related genes, which were preliminarily validated by protein localization and gene expression results. Finally, we construct an online machine learning approach for people more convenient to use.

## Materials and methods

### RNA-Seq expression data processing

We process RNA-Seq data from samples from two different groups of six maize photosynthetic mutants (*hcf136, pyg56, pet2, atp4, bsd2, cps2*) under two treatments. Each mutant was treated with high light and low light, and their second leaves were collected and sequenced, total 95 samples. The original sequencing data were aligned to the maize (Zea mays) B73v4 using TopHat2. Then we use Cufflinks to quantify the raw read count in each gene. And we took DESeq2 [8] to analyze the different information between the wild-type sample and the corresponding mutant sample. Get the different information include the average of the normalized counts for all samples(baseMean), log base twofold changes for the condition tested (Log_2_FoldChange), Log_2_FoldChange Standard Error (lfcSE), Wald statistic (stat), Wald test P value for the Log_2_FoldChange estimate (*P* value) and FDR adjusted *P* value.

### Dataset construction

We took MapMan Mercator4 [5] to annotate maize genes. The Mercator 4 program is based on BLAST sequence similarity and protein domains from InterPro and the Conserved Domain Database, which assign genes that do not show high sequence similarity to Arabidopsis or contain no typical protein domains to the category of “not assigned”. This part of genes in maize is our unannotated genes. This program assigns genes to photosystem I, photosystem II, photorespiration and the Calvin cycle are directly related to photosynthesis [6], is our positive label samples. And this program assigns genes to chromatin organization and cytoskeleton organization that were not directly related to photosynthesis, is our negative label samples.

The expression data and different information were connected according to gene id. Then, we built a dataset from these preliminary datasets. This dataset has 333 features. The statistical results of these three datasets are shown in Table 1. In particular, the not assigned parts were used to predict novel genes related to photosynthesis.

**Table 1:** Overview of dataset

Category	Gene number	Example gene
Photosynthesis related genes	220	*PEPC*(*Zm00001d046170*), *Psb29*(*Zm00001d021763*) …
Not related to photosynthesis genes	405	*IDP2451*(*Zm00001d053643*), *Chr111*(*Zm00001d016861*) …
Unannotated genes	9520	*Zm00001d002341*, *Zm00001d012088*…

### Machine learning architecture

In machine learning applications, each algorithm has different performances on different datasets. Therefore, we used two machine learning methods commonly used in Kaggle (the world's largest data science community) as submodels’ methods, including random forests and gradient boosting machines (Catboost, Gradient Boosting Decision Tree and XGBoost). These four submodels are the first layer of our machine learning architecture. The second layer of our machine learning architecture is the voting mechanism [9, 10, 11, 12].

### Voting mechanism

The voting mechanism can obtain the results of each submodel from the first layer and then make judgments and correct the incorrect prediction of a single model to improve the accuracy of the prediction based on the submodel. For example, if three votes think that a gene is photosynthetic related and two votes think that the gene is not photosynthetic related, the voting mechanism will think that the gene is photosynthetic related.

### Cross-validation

We took a five-fold cross-validation strategy to train our models (Fig. 2a). Next, we trained the four submodels on the training set. Finally, the performance of the four submodels was estimated using the test set by the indicator Area Under the Curve (AUC-ROC) and Recall.

## Results

### Overview of the framework

**Fig. 1:**
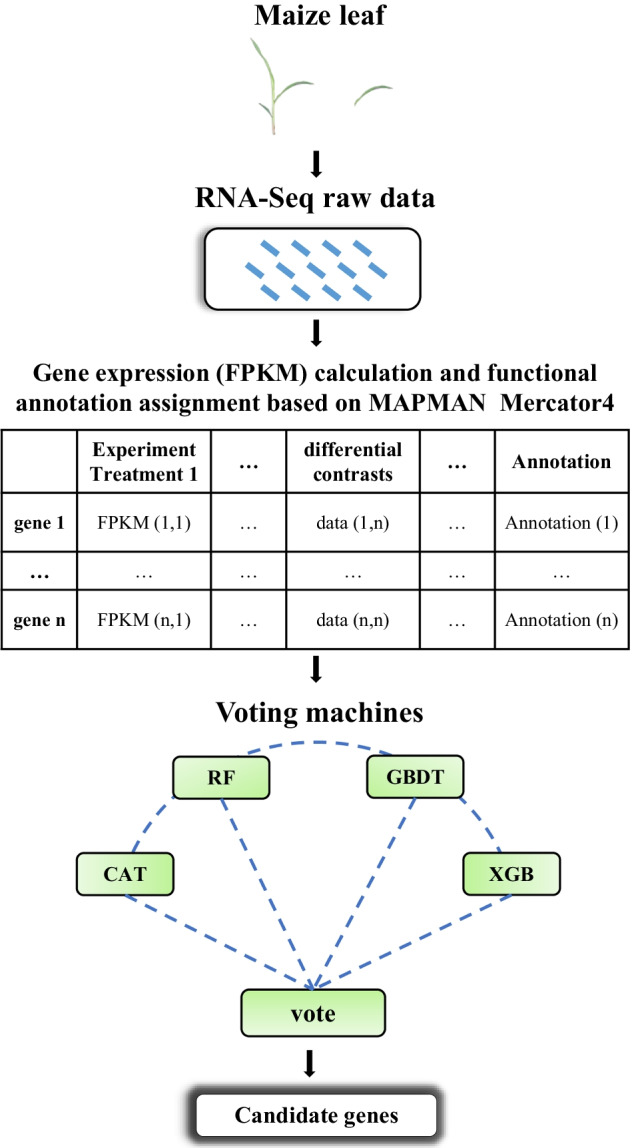
Overview of the PGD framework

We implemented an efficient framework from processing raw RNA-Seq data to obtain the final list of potential photosynthesis-related genes (Fig. 1). To begin this process, we first performed RNA-Seqs on maize leaf two from wild-type (WT) and six photosynthetic mutants (*hcf136*, *pyg56*, *pet2*, *atp4*, *bsd2*, *cps2*) under two treatments (high light and low light), and the raw reads from a total of 72 libraries were processed using Cufflinks and DESeq2 [8] to obtain the Fragments Per Kilobase of exon model per Million mapped fragments (FPKM) expression values and differential contrasts. Then, the expression values and differential contrasts from the different samples were integrated into one file based on gene identification (ID). After that, MapMan Mercator 4 was used to assign genes into different functional categories. Later, we chose 220 genes in the category of photosynthesis as positive training sets, 405 genes that were not assigned to photosynthesis (assigned to chromatin organization and cytoskeleton organization) as negative training sets, and 9520 functional “not assigned genes” as an exploration set to mine putative novel photosynthesis-related genes (Table 1, Additional files 2–4: Tables 1–3). The training set was input into four different supervised machine learning submodels, and the primary results were output. Finally, the primary results from four different submodels were further input into the voting mechanism, and these genes with potential functions related to photosynthesis were selected.

### Performance of the four submodels

**Fig. 2:**
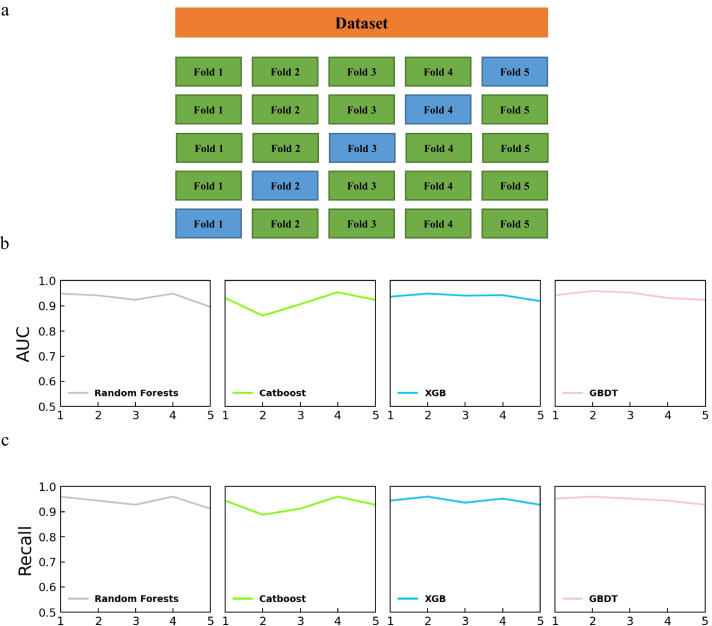
The performance of four sub-models. **a** fivefold cross validation. **b** AUC of sub-models using fivefold cross validation strategy. **c** Recall of sub-models using fivefold cross validation strategy

We observed that a single submodel performed differently in different durations of fold, and the AUC-ROC (Fig. 2b) and Recall (Fig. 2c) was different in four submodels using the same training set. For example, we noticed the highest AUC-ROC score of 0.953 in the fourth fold in Catboost and in the third fold of Gradient Boosting Decision Tree (GBDT). As shown in Fig. 2b, c, single model reported preferences in specific data sets. The model Random Forest had the highest AUC-ROC in the first and fourth folds, while the lowest appears in the fifth fold. The model Catboost had the highest AUC-ROC in the fourth fold, while the lowest appeared in the second fold. The model XGBoost (XGB) and GBDT had the highest AUC-ROC in the second fold and the lowest in the fifth fold. All these results indicated that single model had preferences in specific dataset.

### Construction and performance of the PGD

Because single model has unstable performance in specific dataset, we applied a voting mechanism approach to eliminates the preferences of single machine learning model. Therefore, we constructed an ensemble ML model using a voting scheme. In this scheme, we first trained the four submodels and then voted the results from these four models. We gave three votes to GBDT, the model with the best performance in the previous single submodel test, one vote to Catboost, two votes to XGB and Random Forest. Introducing the voting mechanism of PGD greatly eliminates the preferences of single machine learning model. As shown in Tables 2 and 3, the voting mechanism showed the highest performance in the second, fourth and fifth folds. Even in the second and first fold, the performance of the voting mechanism is less than 0.1 AUC-ROC away from the highest. In general, the mean AUC-ROC and Recall from the voting mechanism was much better than others. Overall, these results indicate that the voting mechanism performance better than a single machine learning model.

**Table 2:** Performance comparison of different methods by AUC-ROC

	Fold1	Fold2	Fold3	Fold4	Fold5
RF	**0.948**	0.941	0.924	0.948	0.896
CAT	0.931	0.862	0.906	0.953	**0.924**
XGB	0.936	0.948	0.940	0.942	0.918
GBDT	0.942	0.959	**0.953**	0.931	**0.924**
VOTE	0.942	**0.964**	0.940	**0.965**	**0.924**

Bold font indicates the highest value in the Fold

**Table 3:** Performance comparison of different methods by Recall

	Fold1	Fold2	Fold3	Fold4	Fold5
RF	**0.960**	0.944	0.928	0.960	0.912
CAT	0.944	0.888	0.912	0.960	**0.928**
XGB	0.944	**0.960**	0.936	0.952	**0.928**
GBDT	0.952	**0.960**	**0.952**	0.944	**0.928**
VOTE	0.952	**0.960**	0.936	**0.968**	**0.928**

Bold font indicates the highest value in the Fold

### Increasing PGD model performance by using RNA-Seq data from multiple photosynthetic mutants

**Fig. 3:**
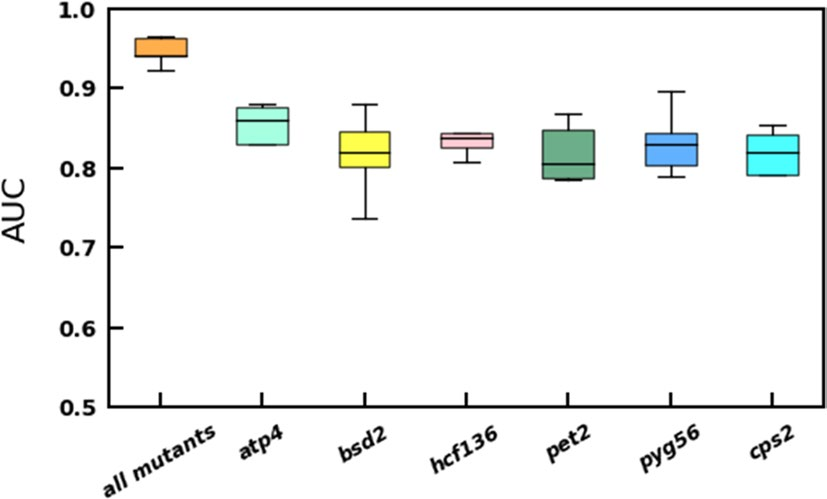
Performance comparison of PGD model using single and multiple photosynthetic mutants

Previous studies have shown that adding related features to data can significantly improve classification performance [13]. Therefore, we tested the performance of the PGD model constructed with RNA-seq from a single photosynthetic mutant and six different types of photosynthetic mutants, including *hcf136, pyg56, pet2, atp4, bsd2* and *cps2* (Fig. 3). The results indicated that in predicting the target genes, the training effect of the dataset constructed from the six photosynthetic mutants was significantly higher than that of the dataset constructed by a single photosynthetic mutant (Fig. 3). Therefore, a large amount of relevant data related to specific research can significantly improve the performance of the model when predicting the target genes.

### Predicting photosynthesis-related genes from function “not assigned” genes using the PGD model

**Fig. 4:**
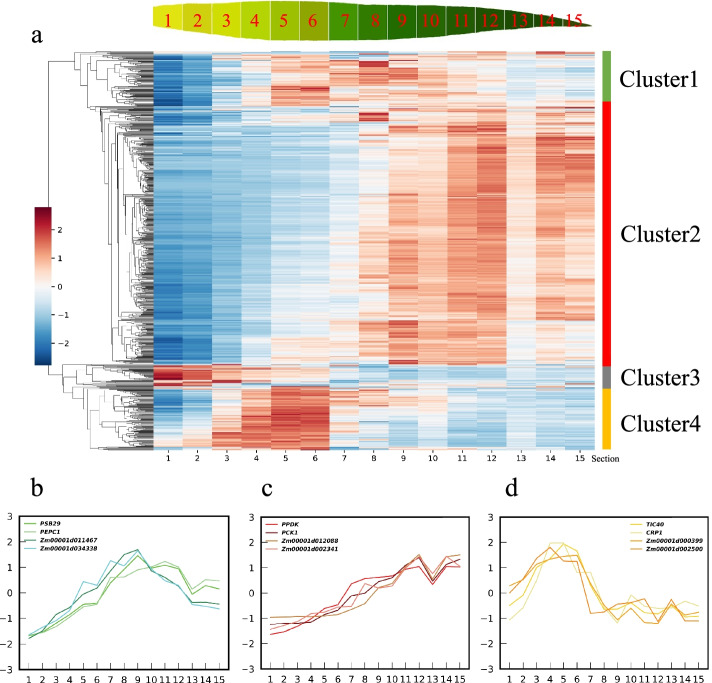
Expression changes of predicted photosynthetic related genes along maize leaf gradient. **a** Hierarchy clustering showing the expression changes of 716 genes along maize leaf sections from base to tip. **b** The expression similarity comparison among predicted genes in cluster 1 and classical photosynthetic related genes. **c** The expression similarity comparison among predicted genes in cluster 2 and classical photosynthetic related genes. **d** The expression similarity comparison among predicted genes in cluster 4 and classical photosynthetic related genes

After the series of tests mentioned above, we retrained the PGD model using the RNA-seq dataset constructed from all six maize photosynthetic mutants to mine the photosynthesis-related genes from the 9520 genes labeled “not assigned” in MapMan Mercator 4. In total, the model predicted 716 genes with a high possibility of participating in the process of photosynthesis in maize (Additional file 3: Table 2). To evaluate whether these 716 predicted genes truly have potential functions in photosynthesis, we checked their expression levels in 15 maize leaf sections along the developmental gradient [14], which also represented a gradient from no photosynthesis (sink, sections 1–3) to chloroplast biosynthesis (source-sink transition zones, sections 4–6) and from low to high photosynthesis, with a slight drop in the leaf tips (source, sections 7–15) of maize leaves. As shown in Fig. 4a, a clear expression trend with four clusters appeared. The genes (15.08%) in Cluster one, e.g., *Zm00001d011467* and *Zm00001d034338*, showed low expression from sections 1–4 (Fig. 4b), and their expression peaked from sections 5–9 and gradually decreased from sections 9–15 (Fig. 4c), similar to the expression profiles of photosystem II subunit29 (*psb29*) and phosphoenolpyruvate carboxylase(*PEPC*) (Fig. 4d), the key genes that participate in photosystem II [15–17], indicating potential functions of these genes related to the chloroplast and photosynthesis functions. Cluster two, with 58.10% of the predicted genes, e.g., *Zm00001d012088* and *Zm00001d002341*, showed low to high expression levels along the leaf gradient, and expression dramatically increased after section 7, similar to the classical maize C4 genes of *PPDK* and *PPCK*, indicating a close link of their functions related to photosynthesis. The expression of the 93 genes in Cluster four (e.g., *Zm00001d000399* and *Zm00001d002500*) increased beginning in Section three, peaked in sections 5–6, and then ceased immediately after section 7, which is highly consistent with the features of genes involved in chloroplast biosynthesis and assembly, such as *Tic40* (translocon Tic40) and *crp1* (chloroplast RNA processing 1) (Fig. 4c) [18]. Although 33 genes (4.6%) appeared to be highly expressed in the sink tissue (sections 1–3), the majority (89.25%) of our predicted genes showed clear expression trends similar to those of genes directly related to the PS (Fig. 4b), indicating a high accuracy of our model in predicting putative photosynthesis-related genes from the “not assigned” category.

We further checked the possible localization of these 716 predicted genes using Target-P v2.0 [19]. Interestingly, 235 of them, which accounted for 32.82% of the total genes, were located in the chloroplast, as predicted by Target-P v2.0, further indicating their potential functions in photosynthesis. Finally, we listed a set of genes whose expression levels increased from the base to tip and were localized in the chloroplast (e.g., *Zm00001d002341*) (Fig. 4d, Additional file 4: Table 3), which we believe are worthy of further research to discover their true biological functions.

### Construct an online machine learning approach

In order to let more people use this approach, we construct an online approach based on google colab. Just upload the form as required (Additional file 1), click on the required steps, and then you can get the gene you are interested in. For example, if you want to find more genes related to sugar synthesis in tomato, you only need to find multiple sugar-related genes in the literature, and then find several genes that are not related to sugar-based on biological knowledge. Through the expression amount generated by RNA-Seq or input the data table you think is important, upload it to the colab according to the attached operation, and then you can find the genes related to sugar synthesis in the gene list of unknown function.

## Discussion

Machine learning methods have been widely used in biology, especially for predicting three-dimensional protein structures [4] and automatically quantifying the phenotypes of crops (e.g., wheat ears) [20]. They have also been used to predict condition-specific regulatory genes in plants [13]. There are 39,498 genes in the maize v41 genome, but according to the most recent MapMan annotation file in Mercator 4, 9520 genes still cannot be assigned to a proper functional category and are labeled “not assigned”, which account for approximately 24.1% of the total genes in maize. Therefore, it is necessary to use a machine learning approach to predict the functions of these genes.

Plant photosynthesis provides both carbohydrates and oxygen to living organisms on our planet. Therefore, it is essential to explore the genes that control photosynthesis. In our study, we implemented an ensemble based ML methods using a majority voting scheme referred to as PGD to predict novel photosynthesis-related genes in maize using RNA-seq expression data from different types of photosynthetic mutants (Fig. 1). In PGD, the genes belonging to the functional category of “photosynthesis” in MapMan Mercator 4 that directly participate in photosynthesis were chosen as the positive training set, and genes included in the functional categories of chromatin organization and cytoskeletal organization that showed no relationship with photosynthesis were chosen as the negative training set and were supplied to the ensemble machine learning models (Fig. 2). The PGD model could select potential photosynthetic genes from 9520 genes that were labeled “not assigned”. We performed cross-validation for PGD, and the evaluation results showed that the features of the integrated output can predict photosynthetic genes accurately (AUC-ROC = 0. 9649). Our results highlighted several important discoveries that could provide new insights into candidate gene mining in plants. First, the voting mechanism performs more stably than the single submodels of Catboost, GBDT, XGB and Random Forest (Fig. 3). Second, datasets that were constructed from all six photosynthetic mutants significantly increased model performance relative to datasets based on single mutants (Fig. 4). Third, our PGD model predicted 716 novel photosynthesis-related genes from 9520 “not assigned” genes (Fig. 4, Additional files 3, 4: Tables 2, 3). This prediction is highly reliable since most of the predicted genes showed similar expression patterns to photosynthetic genes and genes involved in chloroplast biosynthesis, and 235 of them were directly localized in the chloroplast, as predicted by the TargetP program.

Our PGD approach predicted photosynthesis-related genes with high accuracy based on gene expression levels. With the rapid improvement of second-generation sequencing, increasing amounts of RNA-Seq data from wild-type and mutant plants under various treatment conditions have been generated. Our PGD approach could be expanded to any species to help identify novel genes related to a specific functional category, and further biological experiments performed on these candidate genes could help researchers gain more knowledge in their field of interest.

## Conclusions

In summary, our PGD approach integrates four powerful machine learning models, which can stably maintain the detection performance. We predicted 716 photosynthesis-related genes from the “not assigned” category of maize MapMan annotation. The protein localization prediction (TargetP) and expression trends of these genes from maize leaf sections indicated that the prediction was reliable and robust. Our research provides a new approach for studying the functional categories of genes.

## Supplementary Information

**Additional file 1:** Guidance of the online approach.

**Additional file 2:** PGD predicted photosynthesis-related genes.

**Additional file 3:** The Target-P result of PGD predicted photosynthesis-related genes.

**Additional file 4:** Predicted photosynthesis-related genes' protein localized in chloroplast by Target-P.

## Data Availability

RNA-seq data for maize photosynthetic mutants were deposited in the NCBI with the accession code of PRJNA723491. Code to reproduce is available at GitHub repository (https://github.com/supermanwasd/PGD-A-Machine-Learning-based-Photosynthetic-related-Gene-Detection-approach). Online approach is available at google colab (https://colab.research.google.com/drive/1PaYfy2ZpSrKGvrX4D-0i329u-rfGp0WX?usp=sharing).
