# Supplementary material for: PGD: a machine learning-based photosynthetic-related gene detection approach
Source: BMC Bioinformatics. 2022 May 17;23:183. doi: 10.1186/s12859-022-04722-x (PMC9112524; doi:10.1186/s12859-022-04722-x)
Supplement: Supplementary file 1 — Additional file 1: Guidance of the online approach. [file 12859_2022_4722_MOESM1_ESM.docx]

Guidance of the online approach

1. Prepare two tables in csv format.

2. The two tables are named Dataset_an.csv and Dataset_unan.csv, respectively.

3. The table named Dataset_an.csv must have two columns named “GeneID” and “ps”, and the “ps” column is the target column. The “ps” column can only be “1” and “0”, where “1” represents a gene with a known function, “0” represents a known gene that has no clear connection with this function, and the more genes in the two categories, the more accurate the effect. The middle column is the expression quantity, which can be named arbitrarily. The middle column also determines the performance of the model. The more middle columns, the greater the difference, the better.

4. The table named Dataset_unan.csv must have a “GeneID” column and the expression column must be consistent with the table named “Dataset_an.csv”. The genes in the table are candidate genes, and the model will select genes related to the target function.

5. Open link <https://colab.research.google.com/drive/1PaYfy2ZpSrKGvrX4D-0i329u-rfGp0WX?usp=sharing>. And you will see this interface (Fig 1).


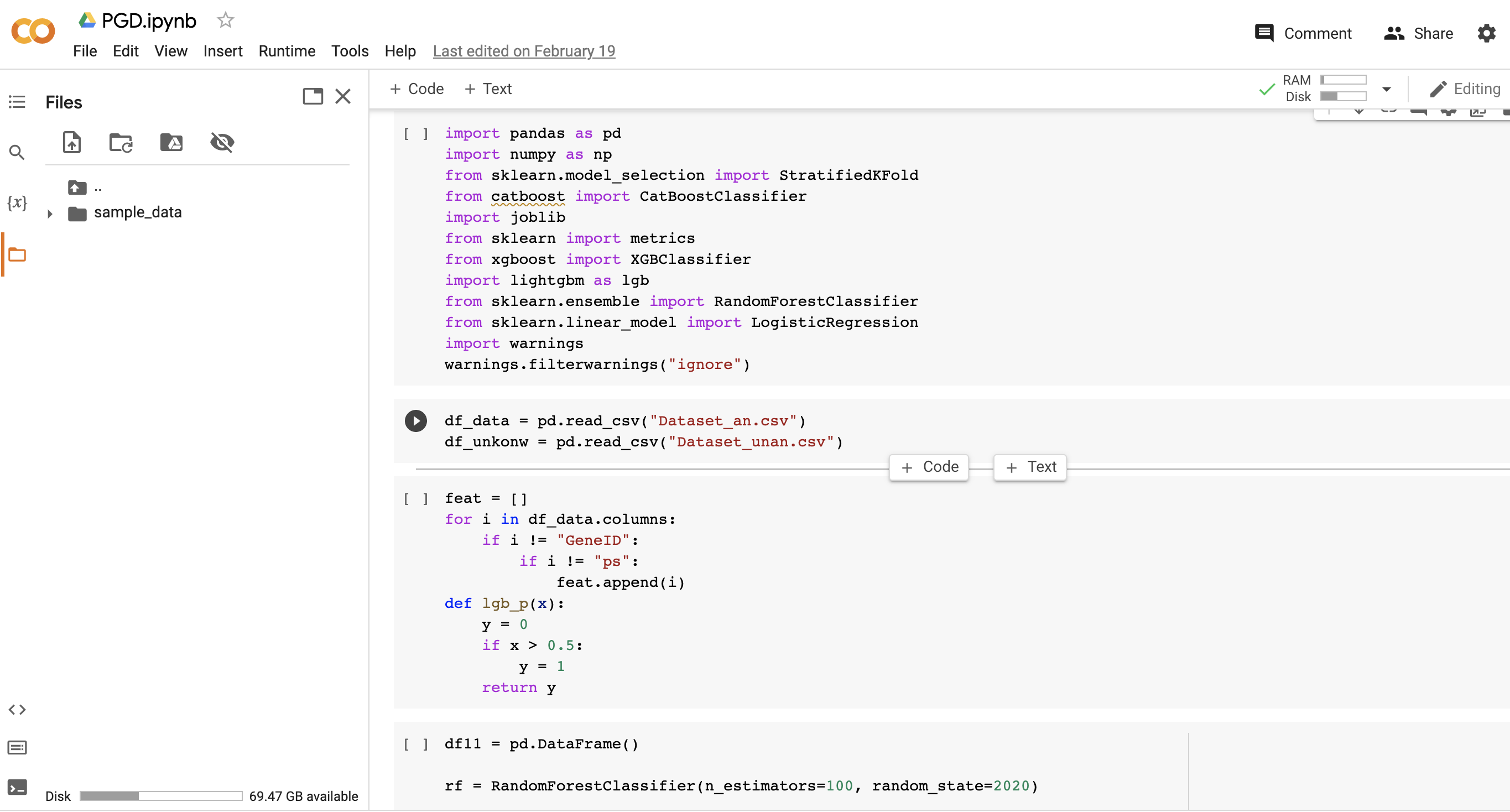


Click this link to upload the two tables.

**Figure 1** The interface of colab.

6. Referring to the arrow in Fig 2, click Run to run all the codes.
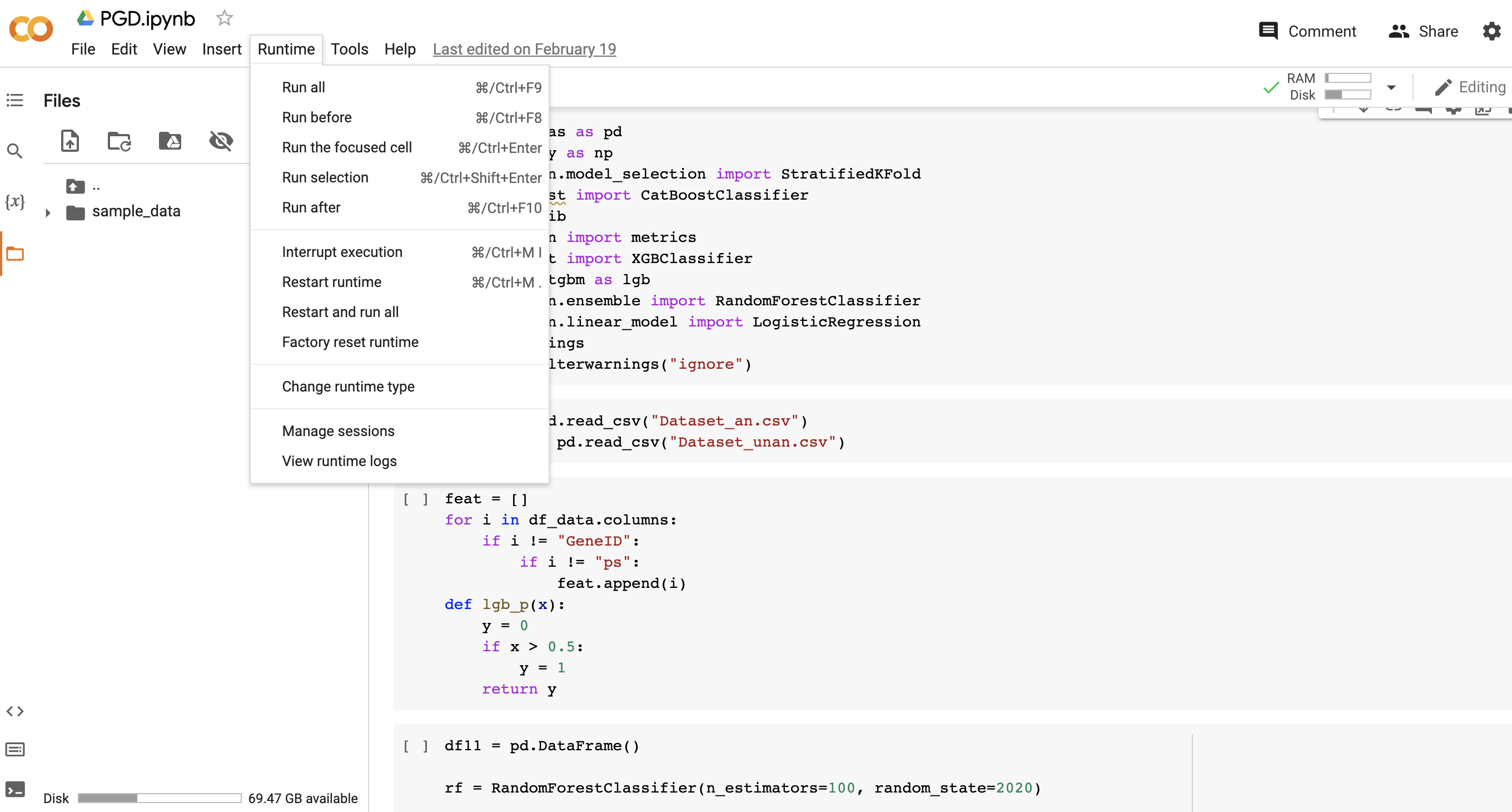


Click this link to run.

**Figure 2** The guide of online step.

7. Download the output result (Fig 3).


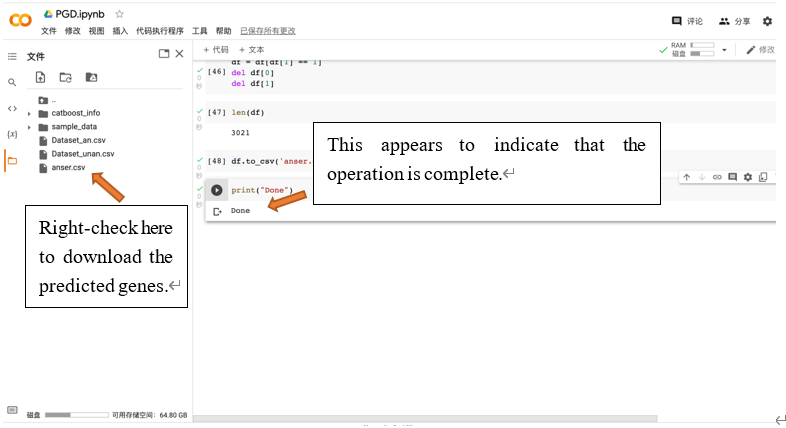


**Figure 3** The guide of download result.
